# Cardiovascular markers of inflammation and serum lipid levels in HIV-infected patients with undetectable viremia

**DOI:** 10.1038/s41598-018-24446-4

**Published:** 2018-04-17

**Authors:** Klaudija Višković, Snježana Židovec Lepej, Ana Gorenec, Ivana Grgić, Davorka Lukas, Šime Zekan, Anja Dragobratović, Maja Trupković, Josip Begovac

**Affiliations:** 1 University Hospital for Infectious Diseases, Zagreb, Croatia; 2 Faculty of Science, University of Zagreb, Zagreb, Croatia; 3 School of Medicine, University of Zagreb, Zagreb, Croatia

## Abstract

Patients successfully treated for HIV infection still have an increased risk for cardiovascular morbidity and mortality, which might be related not only to traditional risks, but also to inflammation and dyslipidemia. We examined the relationship of serum lipid levels with plasma biomarkers of inflammation using a composite inflammatory burden score (IBS) based on individual (>75^th^ percentile) measurements from the following seven markers: CD40L, tPA, MCP-1, IL-8, IL-6, hCRP and P-selectin. IBS was categorized as 0 (none of the biomarkers >75^th^ percentile), 1, 2 and 3 or more scores. Correlations between the IBS and lipid parameters were examined by ordered logistic regression proportional odds models to estimate the odds of more elevated biomarkers. 181 male patients with undetectable HIV-viremia were included into the study. In the multivariate model, a one-unit increase (mmol/L) of total cholesterol and triglycerides was associated with a 1.41-fold (95% CI, 1.13–1.76) and 1.37-fold (95% CI, 1.18–1.60) increased odds of having a greater IBS, respectively. Those with an IBS score ≥1 compared to none had 2.14 (95% CI, 1.43–3.20) higher odds of having a one-unit increased total cholesterol/HDL-cholesterol ratio. In successfully treated HIV-infected persons dyslipidemia was associated with inflammation.

## Introduction

Cardiovascular diseases are important causes of morbidity and mortality in persons infected with human immunodeficiency virus type 1 (HIV-1)^1–3^. The risk of cardiovascular abnormalities (mainly coronary artery disease, CAD) in HIV-infected persons is estimated to be 1.5-2-fold higher compared to uninfected persons^1^. Underlying mechanisms leading to these observations are complex and multifactorial^1^. Traditional risk factors play a role as well as HIV infection *per se* and antiretroviral therapy (ART)^1,4,5^.

Persistent immune activation and systemic inflammation, plays a central role in the pathogenesis of HIV-disease, and also contributes to the increased risk of CAD in both untreated and successfully-treated patients. It is also considered to be related to earlier presentation of CVD in HIV-infected patients compared with the general population^6,7^. Suppression of viral replication by antiretroviral drugs does not completely abrogate viral replication, particularly in viral reservoirs, resulting in persistent residual inflammation and increased risk for the development of CVD even in successfully treated patients^2,8^.

Evaluation of inflammatory biomarkers in the context of CVD risk in HIV-infected persons is challenging due to a variety of methodological issues including differences in study designs, choices of inflammatory biomarker panels as well as selection of clinically relevant outcomes (occurrence of myocardial infarction, cardiac death or the use of surrogate markers of CVD such as carotid intima-media thickness CIMT and markers of arterial stiffness)^9^_._

Vos *et al*.^9^ performed a systematic literature review on the associations between inflammatory biomarkers and CVD or CIMT in HIV-infected persons that included 33 original datasets and analysis of 48 immune markers. Increased concentrations of C-reactive protein (CRP), D-dimers and interleukin-6 (IL-6) in the serum of HIV-infected patients were the only biomarkers significantly associated with the occurrence of CVD^9–13^. Additionally, current literature data do not provide consistent evidence on the association between immune markers of inflammation, endothelial activation or coagulation as well as other biological response modifiers including growth factors and chemokines with CIMT^9^. Since these results are, in part, related to the heterogeneity of study design and selection of biomarkers, further research on the association between CVD and inflammatory biomarkers, particularly in the context of antiretroviral therapy, are needed.

Lipid abnormalities in untreated HIV-infected persons are characterized by hypertriglyceridemia, low HDL-cholesterol and low LDL-cholesterol^14^ whereas after initiation of antiretroviral therapy (ART) LDL and total cholesterol increase^15^. In Croatia, the prevalence of dyslipidemia in treated patients was high (64.6%)^16^. Levels of oxidized LDL and HDL in HIV-infected persons have been associated with some markers of immune activation^8,17^.

The aim of this study was to analyze the possible association between serum lipid levels and plasma biomarkers of inflammation using a composite inflammatory burden score (IBS) from the following seven markers of inflammation: CD40L, tPA, MCP-1, IL-8, IL-6, hCRP and P-selectin in successfully treated HIV-infected patients. In addition to widely studied inflammatory markers (CRP and IL-6), we also investigated CD40L, tPA, MCP-1 and IL-8. Increased concentrations of CD40L have been described in acute coronary syndrome patients and have been linked with the severity of coronary artery disease in patients with acute coronary syndrome (Fong *et al*.; Zhao *et al*.)^18,19^. Altered plasma levels of tPA were shown to be related to carotid intima media thickness in renal transplant recipients (Brzosko *et al*.)^20^. Serum levels of MPC-1, in combination with IL-6, were shown to predict the presence of coronary artery disease and mortality in patients undergoing coronary angiography (Tajfard *et al*.)^21^. Elevated levels of plasma IL-8 represent an independent predictor of long-term all-cause mortality in patients with acute coronary syndrome (Cavusoglu *et al*.)^22^. To our knowledge, this is the first study investigating the association of a panel of markers of inflammation with serum lipid levels in successfully treated HIV-infected men.

## Methods

### Study design and patients

This was a cross-sectional study. Subjects were selected among consecutive HIV-infected males ≥18 years of age with undetectable viral load (<50 copies/ml of HIV-1 RNA) receiving clinical care at the University Hospital for Infectious Diseases (UHID), Zagreb, Croatia in the period between January 2012 to March 2013. Informed consent was obtained from all participants. The study was approved by the Ethics committee of UHID on December 15th 2011 and the research was performed in accordance with the Declaration of Helsinki.

A standardized abstraction form was created which included information on demographic characteristics, HIV disease characteristics (current CD4+ T-cell count, nadir CD4+ T-cell count, duration of HIV infection, duration of ART, current abacavir use, current use of lopinavir, duration of undetectable viremia), lipids (total cholesterol, HDL-cholesterol, LDL-cholesterol and triglyceride), blood pressure, family history of CVD and diabetes, smoking, hepatitis B and C coinfection. The data were extracted from the UHID HIV-1 electronic database.

### Measurements

Biomarkers selected for this study included soluble CD40 ligand (sCD40L), IL-6, IL-8, monocyte chemoattractant protein-1 (MCP-1), soluble P-selectin (sP-selectin) and tissue plasminogen activator (t-PA). Quantification of cardiovascular risk biomarkers in the plasma of HIV-infected patients was performed by using bead-based flow cytometry and Human Cardiovascular 6plex Kit FlowCytomix (eBioscience, San Diego, California, USA) according to the manufacturer’s instructions. To detect each of 6 analytes, the immunoassay utilized beads coated with specific antibodies that were mixed with 25 µl of plasma samples. After incubation, a biotin-conjugated secondary antibody was added and the analyzed cardiovascular risk markers were detected by using streptavidin-phycoerithrin that binds to the biotin conjugate and generates fluorescent signals. Quantification of cardiovascular risk markers was performed by using Cytomix FC500 flow cytometer (Beckman Coulter, Brea, California, USA) and Forward Scatter (FCS) measurements were collected at 1–8°. To calculate cardiovascular risk marker concentrations, measured results were processed by using FlowCytomixPro software (Beckman Coulter, USA).

Absolute counts of CD4+ T-cells were determined by Cytomics FC500 flow cytometer (Beckman Coulter, Brea, California, USA). Periferal blood was stained with two different combinations of fluorochrome-conjugated antibodies CD45-FITC/CD4-RD-1/CD8-ECD/CD3-PC-5/CD38-PC7 and CD45-FITC/CD56-RD-1/CD19-ECD/CD3-PC-5/HLA-DR-PC7 (Beckman Coulter). Absolute counts of CD4+ T-cells were determined by using Flow-Count Fluorospheres (Beckman Coulter). Flow cytometric analysis of peripheral blood lymphocyte populations (including CD4+ T-cell counting) and biomarker quantification were performed on the same day. Flow cytometric analysis of peripheral blood lymphocyte subpopulations and biomarker quantification were performed immediately following sample collection. The total cholesterol, HDL-cholesterol, LDL-cholesterol and triglycerides were determined on Beckman Coulter AU analyzer immediately following sample collection.

C-reactive protein was quantitatively determined in participant’s serum on Beckman Coulter AU analyzer, immediately following sample collection. Immune complex formed in solution scatter light in proportion to their size, shape and concentration. Turbidimeters measure this reduction of incidence light due to reflextion, absorption, or scatter. In this procedure, the measurement of the rate of decrease in light intensity transmitted (increase in absorbance) through particles suspended in solution is the result of complexes formed during the antigen-antibody reaction.

Ultrasound measurement of intima media thickness (IMT) was performed at the same visit when the plasma blood samples were collected and were performed and read by a single, experienced radiologist, using Samsung Medison UGEO H60 ultrasound machine, using electronic calipers. Previously published protocol was used for measurement of the right and left common carotid artery (CCA) IMT and plaque presence^23^. The average value for the right and left CCA was used in the analysis. Subclinical atherosclerosis was considered present if IMT was ≥0.9 mm and/or presence of plaque. Atherosclerotic plaque was defined according to the Mannheim intima-media thickness consensus^24^. IMT measurements of all participants were performed manually and read by a specialist of radiology and appropriate images were recorded on the thermal paper with patient’s code number and saved at the secure place.

### Calculations of risk scores

Ten-year risks for CVD were calculated using the Framingham equation as published by Anderson *et al*.^25^ and were categorized as follows: <10%: low risk, 10–20%: moderate risk, ≥20%: high risk. We applied the original Data collection on Adverse effects of anti-HIV Drugs study (DAD) risk equation exactly as published^26^; a 5-year CVD risk >5% was considered high. DAD risk equation takes into account age, gender, total and HDL cholesterol, smoking status (current or past), blood pressure, history of diabetes, family history of CVD, and exposure to indinavir, lopinavir and abacavir. They were classified as having low (<1%), moderate (1 to 5%), high (5 to 10%) or very high (>10%) risk of CHD over a 5-year period. Subclinical atherosclerosis was present in 58 (32%) of patients.

Plasma inflammatory biomarkers >75th percentile were considered elevated and an inflammatory burden score (IBS) was constructed from the seven biomarkers. A score of one was assigned to a value >75th percentile of an individual biomarker, and a composite score was constructed as the presence of zero, one, two, or three or more elevated biomarkers. It has been suggested that because of multiple comorbidity contributing to inflammation, using a composite measure of inflammation may be more appropriate in the HIV-infected populations^27^. Furthermore there are no thresholds associated with clinical events for many of biomarkers and using multiple biomarkers potentially reduces the variability (intra and inter-person) inherent in measuring and analyzing individual biomarkers. Associations between biomarker quartiles and parameters of HIV control as well as clinical endpoints such as mortality have also been described^28,29^.

### Statistical methods

Values are described as frequencies and percentages or medians with first and third quartile (Q1, Q3). We used the chi-square or Fishers exact test for comparison of categories and the Wilcoxon-Mann-Whitney test for comparison of continuous variables. Correlations between the IBS and lipid parameters were examined using Spearman’s Rho. The strength of the correlation was considered as follows: very weak (0.00–0.19), weak (0.20–0.39), moderate (0.40–0.59), strong (0.50–0.79) and very strong (0.80–1.00). Ordinal (proportional odds) logistic regression analysis was done to study the variables associated with the presence of the four categories of IBS (0, 1, 2, ≥3). The main predictors were lipid measurements. Potential variables for multivariable analysis were screened by bivariate analysis and our candidate variables for the multivariable models were those with a P-value < 0.15: age, cholesterol, triglycerides, total cholesterol to HDL-cholesterol ratio, nadir CD4 cell count, prior clinical AIDS, current use of abacavir, carotid intima media thickness, and CVD risk scores (DAD 5-year CVD and Framingham 10-year CVD). Several combinations of variables were not included into multivariable models because of collinearity concerns (cholesterol, triglycerides and total cholesterol to HDL-cholesterol ratio; nadir CD4 cell count and prior clinical AIDS; carotid intima media thickness and age). Because only seven patients were positive for hepatitis C antibody this variable was also not included into the multivariable model. The DAD 5-year CVD risk and the Framingham 10-year risk for CVD scores were also not included into our multivariable models because those scores are also derived from values of individual variables (age and cholesterol) already included into the models. The score test was used to test the proportional odds assumption. The ratio of total cholesterol to HDL-cholesterol showed evidence of violation of the proportional odds assumption, so a partial proportional odds multivariable model was fit with this variable. For continuous variables, the linearity assumption was checked by the Box-Tidwell method, it was found to be violated for the cardiovascular scores. Statistical analyses were performed with the SAS version 9.4 (SAS Institute Inc., Cary, North Carolina, USA). A two-tailed P < 0.05 was considered presenting a significant association.

## Results

### Baseline characteristics

A total of 181 male patients were included into the study. Median age of the patients was 46.7 (Q1-Q3, 39.9–55.0) years and median body mass index (BMI) was 24.9 (Q1-Q3, 23.4–27.0). The majority of patients 118/181 (65.2%) were men who have sex with men (MSM) whereas 24.9% (45/181) patients were heterosexuals (Table 1). Current use of lipid lowering drugs was noted in 32/181 patients (17.7%).

**Table 1:** Sociodemographic and HIV-related Characteristics of 181 Male Patients with Undetectable Viremia According to Elevated Biomarkers.

Variables	At least one elevated biomarker			P value
	Yes (n = 130)	No (n = 51)	Total (n = 181)	
Age, years (median: Q1, Q3)	48.1 (41.3, 55.5)	44.5 (36.8, 54.9)	46.7 (39.9, 55.0)	0.068
Body weight, kg (median: Q1, Q3)	80.3 (72.0, 87.0)	78.0 (71.0 87.0)	80.0 (72.0, 87.0)	0.935
Body mass index (median: Q1, Q3)	24.8 (23.5,27.2)	25.0 (23.1,26.8)	24.9 (23.4,27.0)	0.871 s
**HIV transmission (n, %)**				
Heterosexual	32.0 (24.6)	13.0 (25.5)	45.0 (24.9)	0.707
Intravenous drug use	5.0 (3.8)	1 (2.0)	6.0 (3.3)	
Men who have sex with men	86.0 (66.2)	32.0 (62.7)	118 (65.2)	
Other/unknown	7.0 (5.4)	5.0 (9.8)	12.0 (6.6)	
History of clinical AIDS (n, %)	84 (64.6)	40 (78.4)	124 (68.5)	0.072
Hepatitis B antigen positivity, (n,%)	6 (4.6)	1 (2.0)	7 (3.9)	0.405
Hepatitis C antibody positivity (n, %)	7 (5.4)	1 (2.0)	8 (4.4)	0.445
Duration of known HIV-infection, years (median: Q1, Q3)	8.0 (4.0, 12.0)	6.0 (3.0, 10.0)	7.0 (4.0, 11.0)	0.088
Nadir CD4 count per μL (median: Q1, Q3)	110.0 (29.0, 201.0)	136.0 (55.0, 229.0)	116.0 (30.0, 208.0)	0.170
Current CD4 cell count per μL (median: Q1, Q3)	571.0 (389.0, 752.0)	535.0 (381.0, 719.0)	553.0 (389.0, 729.0)	0.534
Duration of ART years (median: Q1, Q3)	6.1 (2.9, 9.0)	4.5 (1.9, 8.8)	5.2 (2.6, 8.8)	0.182
Current use of lipid lowering drugs (n, %)	25 (19.2)	7 (13.7)	32 (17.7)	0.382
Current abacavir use (n, %)	73 (56.2)	22 (43.1)	95 (52.5)	0.115
Current lopinavir use (n, %)	39 (30.0)	14 (27.5)	53 (29.3)	0.735

Q1, first quartile; Q3, third quartile.

Median duration of known HIV infection in the patients was 7.0 (Q1-Q3, 4.0–11.0) years and the median current CD4 + T-cell count was 553/μL (Q1-Q3, 389–729). The patients were mainly treated with two nucleoside reverse transcriptase inhibitors (NRTI) plus one non-NRTI (NNRTI) (n = 100, 60.8%) or two NRTI plus lopinavir (N = 50, 27.6%). Median duration of ART was 5.2 (Q1-Q3, 2.6–8.8) years. At the time of analysis, 52.5% (92/181) patients were treated with abacavir. The prevalence of HBsAg was 3.9% (7/181 patients) and anti-HCV antibodies were detectable in 4.4% of patients (8/181) (Table 1).

The patients were also classified according to the results of biomarker quantification into two groups: 130 with at least one elevated biomarkers and 50 patients without elevated biomarkers. The differences in the sociodemographic and HIV-related characteristics in the two patient groups were not statistically significant at a P value level of 0.05, however, age was greater, the duration of HIV infection was longer in persons with at least one elevated biomarker and a history of clinical AIDS was somewhat more frequently found in persons with no elevated biomarker (Table 1).

### Cardiovascular risk and inflammatory biomarkers

Patients with at least one elevated biomarker had increased CHD risk assessed by DAD 5-year algorithm (median 3.8, Q1-Q3 2.4–7.6) compared with patients without elevated biomarkers (median 2.7, Q1-Q3 1.1–5.8, P = 0.005) (Table 2). Similarly, significantly increased CVD risk assessed by Framingham 10-year score was observed in patients with at least one elevated biomarker (median 12, Q1-Q3, 6.5–19.8 vs. median 7.2, Q1-Q3, 3.8–16.6. P = 0.003). There was no statistically difference in the median value of IMT between the two patient groups (median 0.7, Q1-Q3 0.6–0.8 vs. median 0.6, Q1-Q3, 0.5–0.8, P = 0.061).

**Table 2:** Cardiovascular risks scores and inflammatory biomarkers in 181 male patients according to elevated biomarkers.

Variables	At least one elevated biomarker			P value
	Yes (n = 130)	No (n = 51)	Total (n = 181)	
DAD 5-year CHD score (median: Q1, Q3)	3.8 (2.4, 7.6)	2.7 (1.1, 5.8)	3.6 (2.0, 7.2)	0.005
DAD 5-year CVD score (median: Q1, Q3)	4.6 (3.0, 9.1)	3.5 (1.5, 7.3)	4.4 (2.5, 8.4)	0.009
Framingham 10-year CVD score (median: Q1, Q3)	12.0 (6.5, 19.8)	7.2 (3.8, 16.6)	10.8 (5.7, 19.2)	0.003
Framingham 10-year CVD score > 20% (n,%)	32 (24.6)	10 (19.6)	42 (23.2)	0.473
CD40L, pg/ml (median: Q1, Q3)	1132 (554, 2196)	846.8 (578, 1147)	933.6 (554, 1862)	0.004
MCP-1 pg/ml (median: Q1, Q3)	583.0 (459, 737)	449.8 (398, 512)	527.7 (428, 672)	<0.001
IL-8, pg/ml (median: Q1, Q3)	7.2 (3.0, 14.3)	2.6 (0.0, 8.6)	6.1 (1.5, 11.1)	<0.001
IL-6, pg/ml (median: Q1, Q3)	3.4 (1.2, 6.5)	0.9 (0.0, 3.0)	2.4 (0.6, 5.0)	<0.001
hCRP mg/L (median: Q1, Q3)	2.2 (1.0, 5.7)	1.0 (0.6,1.8)	1.8 (0.8, 4.2)	<0.001
P-selectin, ng/ml (median: Q1, Q3)	203.23 (162, 269)	183.6 (130, 216)	200.1 (156, 256)	0.014
Carotid intima media thickness, mm (median: Q1, Q3)	0.7 (0.6, 0.8)	0.6 (0.5, 0.8)	0.7 (0.6, 0.8)	0.061
**Inflammatory burden score (n, %)**				
0		51 (28.2)	51 (28.2)	
1	45 (24.9)		45 (24.9)	
2	48 (26.5)		48 (26.5)	
3	20 (11.0)		20 (11.0)	
4	11 (6.1)		11 (6.1)	
5	6 (3.3)		6 (3.3)	

DAD, Data collection on Adverse effects of anti-HIV Drugs study; CHD, coronary haert disease; CVD, cardiovascular disease; IL, interleukin; MCP, monocyte chemoattractant protein; hCRP, high sensitivity C-reactive protein; Q1, first quartile; Q3, third quartile.

Significantly higher concentrations of CD40L (median 1132 vs. 846 pg/mL), MCP-1 (583.vs. 449.8 pg/mL), IL-8 (7.3 vs. 2.6 pg/mL), IL-6 (3.4 vs. 0.9 pg/mL), hCRP (2.2 vs. 1.0 mg/L), and P-selectin (203.2 vs. 183.6 ng/mL) were observed in patients with at least one elevated biomarker compared with patients without increased biomarkers (P = 0.004, P < 0.001, P < 0.001, P < 0.001, P < 0.001 and P = 0.014, respectively) (Table 2). A total of 85 patients (47.0%) had the IBS score ≥ 2 whereas an IBS score ≥ 3 and ≥ 4 was present in 37 (20.4%) and 17 (9.4%) patients respectively (Table 2).

### Association between biomarkers of inflammation and lipid levels

**Figure 1:**
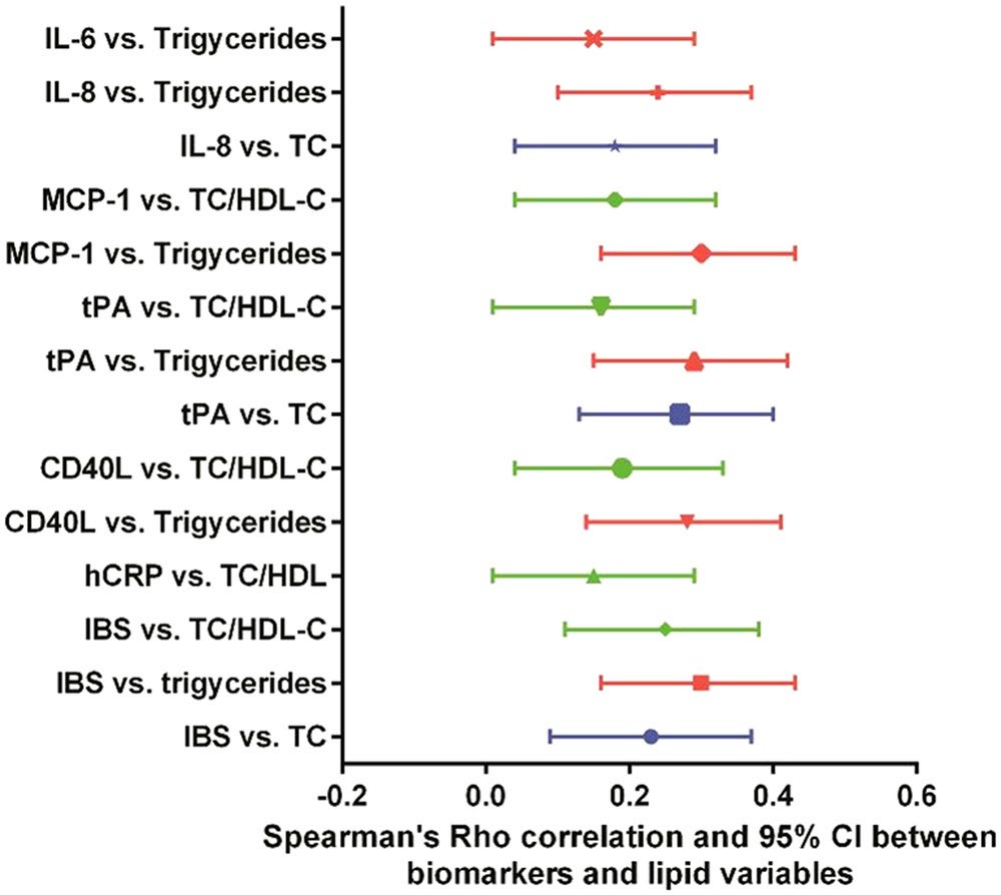
Spearman’s Rho correlation and 95% CI between biomarkers and lipid variables. IL, interleukin; MCP, monocyte chemoattractant protein; TC, total cholesterol; HDL-C, High-density lipoprotein cholesterol; tPA, Tissue plasminogen activator; CD40L, CD40 ligand; hCRP, high sensitivity C-reactive protein; IBS, inflammatory burden score.

There was a significant correlation between the IBS and serum cholesterol (Rho = 0.23, 95% CI, 0.09–0.37, P = 0.002), triglycerides (Rho = 0.30, 95% CI, 0.16–0.42, P < 0.001) and cholesterol/HDL-cholesterol ratio (Rho = 0.25, 95% CI 0.11–0.38, P < 0.001) (Fig. 1).

The results of bivariate ordinal proportional odds logistic regression model is presented on Table S1. In the multivariable model, a one-unit increase (mmol/L) of cholesterol and triglycerides was associated with a 1.41-fold (95% CI, 1.13–1.76) and 1.37-fold (95% CI, 1.18–1.60) increased odds of having a greater IBS, respectively (Table 3). Because of the violation of proportional odds assumption, a partial proportional odds model was fitted for the ratio of total cholesterol to HDL-cholesterol. This model also suggested that a greater IBS score was associated with a higher cholesterol/HDL-cholesterol ratio (Table 3). The comparison of the high IBS (≥3) versus all other had an odds ratio of 1.28 (95% CI, 0.87–1.90, P = 0.211) and the comparison of two or more IBS scores versus one or none had an odds ratio of 1.78 (95% CI, 1.23–2.61, P = 0.001) per one unit of increase in the total cholesterol/HDL-cholesterol ratio.

**Table 3:** Factors related to elevated (>75th percentile) inflammatory burden scores (IBS) on multivariable analysis.

**Characteristics**	Model 1		Model 2		Model 3^a^	
	Odds ratio and 95% CI	P-value	Odds ratio and 95% CI	P-value	Odds ratio and 95% CI	P-value
Current use of abacavir, no versus yes	0.68 (0.39–1.17)	0.165	0.68 (0.40–1.18)	0.172	0.61 (0.35–1.01)	0.074
Past history of clinical AIDS, yes versus no	1.72 (0.96–3.09)	0.069	1.71 (0.95–3.08)	0.074	1.77 (0.99–3.16)	0.056
Age, per 10 years	1.26 (0.96–1.65)	0.095	1.29 (0.98–1.69)	0.069	1.36 (1.04–1.79)	0.026
Total cholesterol, per one mm/L	1.41 (1.13–1.76)	0.003	—	—	—	—
Triglycerides, per one mm/L	—	—	1.37 (1.18–1.60)	<0.001	—	—
Total cholesterol/HDL cholesterol ratio, per one unit^b^	—	—	—	—	2.14 (1.43–3.20)	<0.001

The ordinal proportional odds logistic regression model estimates the odds of having a higher IBS. IBS, inflammatory burden score.

^a^A partial proportional odds model.

^b^Comparison of 1 or more IBS to none.

## Discussion

Successfully treated HIV-infected patients still have an increased risk for cardiovascular morbidity and mortality, which is thought to be related not only to traditional risk factors, but also to inflammation and dyslipidemia induced by HIV and/or antiretroviral therapy. The results of our study showed a significant association between the expression of inflammatory biomarkers and serum levels of cholesterol and triglycerides as well as with the cholesterol/HDL-cholesterol ratio in virologically-suppressed HIV-infected patients treated for a median of 5.2 years. However, the correlation of biomarkers of inflammation with lipids was generally weak (Fig. 1. Rho mainly between 0.20 and 0.30).

Inflammation is an important factor associated with the development of atherosclerosis in HIV-infected patients in all stages of disease. Atherosclerotic plaque is characterized by the accumulation of macrophages, smooth muscle cells and lymphocytes^6^. Chronic HIV infection, in combination with ART, induces dysfunction of endothelial cells which leads to the activation of inflammatory response and promotion of local thrombosis which is an important factor in plaque formation^30^.

CIMT has been used as an early marker of atherosclerosis^31^. Several studies suggested an association between HIV-infection and CIMT but opposing results have also been reported^23,32,33^. In our previous study, we reported subclinical atherosclerosis (CIMT ≥ 0.9 mm and/or the presence of ≥1 carotid plaque) more frequently in in older HIV-infected patients compared with HIV-negative controls^23^. CIMT was greater in patients with a higher IBS score (Tables 2 and S1), however the difference did not reach statistical significance (P = 0.061 and P = 0.051 respectively). It is generally unknown what is the natural course of CIMT in HIV-infected patients. In a large longitudinal IMT study (Mangili, *et al*. 2011) of HIV-infected adults the mean CIMT CCA progression was 0.016 mm per year^34^. Taking in consideration this small rate of CIMT changes per year, a difference of 0.01 mm could have some clinical meaning, although not in a short time interval, but over a decade or more. Longenecker *et al*. (2013) failed to show an association between CIMT or the presence of plaque with pro-inflammatory monocyte subsets and serum markers of monocyte activation including soluble CD163 and CD14 in patients from the SATURN-HIV trial^35^.

Hsu *et al*. (2016) analyzed the association between markers of T-cell and monocyte activation, inflammation and coagulopathy with atherosclerosis and mortality in 149 treated HIV-infected patients with undetectable viremia^36^. Independently of traditional cardiovascular factors, higher plasma concentrations of IL-6 and expression of chemokine coreceptor CCR5 on monocytes were associated with atherosclerosis. Additionally, plasma concentrations of IL-6 and CIMT were individually associated with all-cause mortality.

Studies focusing on the possible use of biomarkers of inflammation and coagulation as predictors of cardiac disease and mortality in HIV-infected patients also investigated their possible association with risk stratification scores, most often Framingham and VACS (Veterans Aging Cohort Study) scores. Mooney *et al*. (2015) have shown an association between both Framingham and VACS scores with elevation in biomarkers of inflammation and coagulation including hsCRP, D-dimers, Cystatin C, IL-6 and TNF-α in a cohort of 252 HIV-infected persons that included 13% of women and 75.7% of virologically suppressed patients^7^. Additionally, patients with a higher number of elevated biomarkers had higher mean VACS scores as well as Framingham scores. These results are in concordance with our results showing that HIV-infected persons with at least one elevated biomarker had a significantly higher DAD CHD and CVD risk scores as well as Framingham 10-year scores (Table 2).

Dyslipidemia in HIV-infected persons has been extensively studied in both untreated and treated patients^37^. It has been shown that different ART regimens as well as different drugs from the same ART class appear to be associated with different patterns of lipid metabolism alterations^37^. The development of dyslipidemia involves multiple factors including persistent immune activation and inflammation in HIV-infected persons. The association between increased lipid levels and concentrations of inflammatory biomarkers in HIV-infected persons is proposed to be based on a two-way process in which inflammation increases serum lipid levels that subsequently enhance the inflammatory processes resulting in increased concentrations of biomarkers^8^.

Kelesidis *et al*. (2016) analysed the association between inflammatory biomarkers (including IL-6, hCRP and D-dimers), immune activation and exhaustion and oxidized HDL and LDL in 234 HIV-infected patients prior to and following 96 weeks of ART who achieved undetectable viremia by week 24 and thereafter^38^. The results of their study have shown a positive correlation between levels of HDL-cholesterol and markers of inflammation and immune activation including IL-6 and soluble CD163 suggesting that oxidized lipoproteins may contribute to persistent immune activation in persons on ART. Despite methodological differences between the two studies, our results also show an association between selected biomarkers of inflammation and parameters of lipid metabolism.

Late initiation of ART (e.g. in patients with <200 CD4+ T-cells per μL) is associated with persistently elevated levels of insulin, triglyceride, IL-6 and hsCRP even after three years of undetectable plasma viremia demonstrating long term persistence of systemic inflammation and metabolic disorder even in successfully treated patients^39^. These results are in concordance with our results demonstrating persistently elevated levels of biomarkers in a proportion of HIV-infected patients experiencing long-lasting virological success.

Our study has several limitations. As all of our cohort were men and Caucasians, so our results may not be generalizable to other populations. We have not studied some biomarkers (eg, CD163, TNF alpha) relevant to inflammation in HIV infected persons. CIMT was measured manually using electronic calipers and may reduce accuracy of measurement which could be achieved by automated computerized edge detection, used in some other studies. Finally, because of the cross-sectional nature of study causation cannot be assessed. Nevertheless, we found a positive association of elevated biomarkers of inflammation with elevated total cholesterol and total cholesterol/HDL-cholesterol ratio, and triglycerides in a population of chronically HIV infected patients in the background of virological suppression following ART.

In summary, we studied seven markers of inflammation in virologically suppressed patients on ART. The majority of patients in our study (68%) did not have subclinical atherosclerosis. There was a relatively high proportion of individuals with two or more (47.0%) or three or more (20.4%) elevated (>75^th^ percentile) biomarkers of inflammation. There was also a significant association between biomarkers of inflammation and serum concentration of cholesterol and triglycerides as well as with the cholesterol/HDL-cholesterol ratio. This might imply that HIV induced inflammation contributes significantly to dyslipidemia. However, dyslipidemia might also enhance inflammation, so further studies are needed to clarify the interrelationship of inflammation and dyslipidemia.

## Electronic supplementary material

Supplemental table
