# Supplementary material for: Cardiovascular markers of inflammation and serum lipid levels in HIV-infected patients with undetectable viremia
Source: Sci Rep. 2018 Apr 17;8:6113. doi: 10.1038/s41598-018-24446-4 (PMC5904142; doi:10.1038/s41598-018-24446-4)
Supplement: Supplementary file 1 — Supplemental table [file 41598_2018_24446_MOESM1_ESM.docx]

Supplemental table

| **S1 Table. Factors related to elevated (>75th percentile) inflammatory burden scores (IBS) in bivariate analysis** | | |
| --- | --- | --- |
| Variables | OR 95% CI | P-value |
| Age, per 10 years | 1.3 (1-1.68) | 0.048 |
| Current or former smoking, (yes versus no) | 1.1 (0.64-1.9) | 0.732 |
| Body mass index, per one unit | 0.98 (0.95-1.02) | 0.312 |
| Subclinical atherosclerosis, (no versus yes) | 0.74 (0.42-1.3) | 0.299 |
| Carotid intima media thickness, per 0.1 mm | 1.14 (0.99-1.31) | 0.051 |
| Known duration of HIV infection, per one year | 1.05 (0.98-1.12) | 0.167 |
| Current use of lopinavir, (yes versus no) | 1.26 (0.71-2.23) | 0.437 |
| Current use of abacavir, (yes versus no) | 1.54 (0.91-2.6) | 0.110 |
| Had clinical AIDS, (yes versus no) | 2.02 (1.14-3.57) | 0.016 |
| Nadir CD4 cell count |  |  |
| < 50 versus > 200 per mm3 | 2.31 (1.18-4.55) | 0.015 |
| ≥50 and ≤200 versus > 200 per mm3 | 1.64 (0.86-3.15) | 0.135 |
| Hepatitis C antibody positivity, (yes versus no) | 3.14 (0.85-11.63) | 0.086 |
| Total cholesterol, per one mmol/L | 1.48 (1.18-1.85) | 0.001 |
| Total cholesterol |  |  |
| < 5.2 mmol/l versus > 6.2 mmol/l | 0.42 (0.22-0.80) | 0.009 |
| 5.2-6.2 mmol/l versus > 6.2 mmol/l | 0.66 (0.35-1.23) | 0.188 |
| HDL-cholesterol, per one unit | 1.09 (0.44-2.68) | 0.853 |
| HDL-cholesterol |  |  |
| < 1.0 mmol/l versus >1.6 mmol/l | 1.51 (0.41-5.60) | 0.604 |
| 1.0-1.6 mmol/l versus >1.6 mmol/l | 1.24 (0.61-2.54) | 0.537 |
| Total cholesterol/HDL cholesterol ratio, per one unit^a,b^ | 1.25 (0.85-1.83) | 0.252 |
| Total cholesterol/HDL cholesterol ratio, per one unit^a,c^ | 1.72 (1.23-2.40) | 0.002 |
| Total cholesterol/HDL cholesterol ratio, per one unit^a,d^ | 2.08 (1.41-3.08) | <0.001 |
| LDL-Cholesterol, per one mmol/L | 1.22 (0.91-1.63) | 0.182 |
| LDL-cholesterol |  |  |
| < 2.6 mmol/l versus ≥ 4.2 mmol/l | 0.39 (0.14-1.08) | 0.070 |
| 2.6-3.3 mmol/L versus ≥ 4.2 mmol/l | 0.37 (0.14-1.01) | 0.053 |
| 3.4-4.1 mmol/L versus ≥ 4.2 mmol/l | 0.34 (0.12-0.99) | 0.047 |
| Triglycerides, per one mmol/L | 1.39 (1.2-1.61) | <0.001 |
| Triglycerides ≥1.7 mmol/l, yes versus no | 1.64 (0.89-3.06) | 0.115 |
| Triglycerides |  |  |
| < 1.7 mmol/l versus ≥5.7 mmol/l | 0.17 (0.06-0.50) | 0.001 |
| 1.7-2.2 mmol/l versus ≥5.7 mmol/l | 0.11 (0.04-0.33) | <0.001 |
| 2.3-5.7 mmol/l versus ≥5.7 mmol/l | 0.35 (0.13-0.95) | 0.040 |
| Framingham 10-years CVD risk, per one % | 1.03 (1.01-1.06) | 0.010 |
| Framingham 5-years CVD risk, per one % | 1.05 (1.01-1.1) | 0.017 |
| Framingham CVD 10-years risk categories |  |  |
| High (≥20%) versus low (< 10%) risk | 2.00 (1.04-3.87) | 0.039 |
| Intermediate (10-20%) versus low (<10%) risk | 2.45 (1.30-4.61) | 0.006 |
| DAD 5-years CHD risk, per one % | 1.06 (1.01-1.11) | 0.026 |
| DAD 5-years CVD risk, per one % | 1.05 (1.01-1.09) | 0.023 |
| DAD CHD risk score categories |  |  |
| Low (<1%) versus high > 5% | 0.08 (0.02-0.32) | <0.001 |
| Moderate (1 to 5%) versus high > 5% | 0.67 (0.39-1.17) | 0.158 |
| Hypertension^e^ | 1.1 (0.65-1.87) | 0.714 |

The ordinal proportional odds logistic regression model estimates the odds of having a higher IBS.

^a^A partial proportional odds model.

^b^ Comparison of the high IBS (3) to all others.

^c^ Comparison of 2 or more IBS scores to 1 or none.

^d^ Comparison of 1 or more IBS to none.

^e^ Systolic BP >140 or diastolic > 90 mmHg or use of antihypertensives, (yes versus no).
